# Normal background levels of air and surface mould reserve in English residential building stock: a preliminary study towards benchmarks based on NAHA measurements

**DOI:** 10.14324/111.444/ucloe.000005

**Published:** 2020-03-06

**Authors:** Yasemin Didem Aktas, Morten Reeslev, Hector Altamirano, Neil May, Dina D’Ayala

**Affiliations:** 1 University College London (UCL), Department of Civil, Environmental and Geomatic Engineering (CEGE), Epicentre Research Group, London WC1E 6DE, UK; 2 UK Centre for Moisture in Buildings (UKCMB), University College London, London WC1H 0NN, UK; 3 Mycometer A/S, Dr Neergaards Vej 3, 2970 Hørsholm, Denmark; 4 University College London (UCL), Institute of Environmental Design and Engineering (IEDE), London WC1H 0NN, UK

**Keywords:** mould, surface sampling, active (aggressive or activated) air sampling, NAHA, England, Denmark

## Abstract

This paper reports results obtained from a surface (both visually clean and dirty/dusty surfaces) and active (aggressive or activated) air testing scheme on 140 residential rooms in England, without visible water damage or mould growth, along with a few rooms with visible mould growth/water damage tested for comparison purposes. The aim was to establish normal background levels of mould in non-water-damaged interiors to benchmark a ‘normal’ indoor environment, and in turn when there is a need for further investigation, and, possibly, remediation. Air and surface mould was quantified based on the activity of β-N-acetylhexosaminidase (EC 3.2.1.52; NAHA). The obtained readings showed a log-normal distribution. Ninety-eight percent of the samples obtained from visually clean surfaces were equal to or less than 25 relative fluorescence units (RFU), which is suggested to be the higher bound for the range which can be used as a success criterion for surface cleaning/remediation. Of samples obtained from visually dirty/dusty surfaces, around 98% were below 450 RFU, which is suggested to define the lower-bound for abnormally high levels of mould, rare even on dirty/dusty surfaces. Similarly, around 98% of the air samples were found to have 1700 RFU or below. Values above 1700 RFU are therefore deemed unlikely in a non-problem indoor environment and can be indicative of a possible problem inducing mould growth. The samples with values below 1700 were further divided into three proposed sub-categories. Finally, the obtained RFU values and the suggested benchmarks were compared to those obtained from 17 non-residential indoor environments tested previously in Copenhagen, and the benchmarks that are currently used in Danish national standards, and they were both found to be highly congruent, suggesting that local climate regimes and room functions might not be as influential on indoor mould levels as commonly thought, or that the nuances between England and Denmark in terms of these factors are not strong enough to lead to sizable changes in the typical indoor mould levels in these countries’ building stocks.

## Introduction

Mould growth is one of the most persistent problems affecting the indoor built environment and the UK is among those countries where it is claimed that this problem is especially pronounced [1] with an estimated >1 million properties considered to have a significantly higher than average risk of harm due to seasonal or continuous mould and damp in England alone [2]. Some of the contributing factors have been identified as rising or penetrating damp due to moisture exposure or construction defects, poor thermal performance, inadequate ventilation, overcrowding, lack of proper insulation, disrepair, and fuel poverty [2–6]. However, despite the increasing needs of the remediation and insurance industries for clear definitions, methodologies, and benchmarks, indoor mould growth in buildings is still, to large extent, an area of confusion for academics and practitioners alike. The interpretation of any obtained results is difficult because there are no scientifically established criteria on the acceptable mould levels or compositions, or on the baseline levels for ‘normal’ indoor environments [7, 8]. An already limited number of suggested values for acceptable indoor mould levels are based on institutional consensus or professional judgement rather than concrete data obtained by means of well-established, transparent testing protocols, and are often contradictory.

The differentiation between a ‘problem’ indoor environment and a ‘normal’ indoor environment has been the subject of many scholarly publications. Richards, in his early review [9], defines a damp/problem home as one where there is visible mould, which is in broad agreement with the findings of Hyvärinen et al. [10]. On the other hand, it has been shown that the lack of visible mould does not necessarily mean that mould levels are low and that testing might still be desirable [11, 12]. However, even when data are available via testing, guidance as to when mould levels should be taken as indicative of a need for remediation is still rather superficial. The guidelines either have no specific criteria, or use visible mould growth (or water damage, or musty odours) as the remediation triggering event (e.g. [13, 14]), which are of little use when high mould concentrations have not (yet) manifested in the form of visible mould, when there is no obvious water damage or when the mould is hidden inside cavities.

One approach that is deeply rooted within the research community is to compare indoor and outdoor concentrations and species. It is suggested that when indoor concentrations are higher than outdoor readings by a certain recommended level (which varies from scholar to scholar; see [8]), then indoor fungal contamination should be suspected [15], unless the houses are extremely dusty [16] or there is some other intramural source of mould [17]. This approach, while seemingly robust, has considerable potential to be flawed as it relies on a limited number of readings obtained from outdoors. Outdoor mould levels (and compositions; see [18]) can greatly vary temporally and spatially due to ever-changing wind speed and direction, anthropogenic activity, weather conditions, especially rain [19, 20], and other local physical characteristics such the extent and nature of flora nearby (as pointed out even in guidelines recommending the use of this approach, see [21]), and there is no well-established guidance to specify how many outdoor readings should be taken, at what time, and from where in reference to the tested indoor environment (cf. [22]). Horner et al. [15] suggest that ‘at least 2 and perhaps up to 10% of the total number of samples come from outdoors’, however, in most studies, one outdoor reading is taken (e.g. [23–25]), which can easily lead to some ‘accidental’ values.

Within this framework, this study aims to establish normal background levels of mould in order to improve the decision-making process as to when a given indoor environment may be in need for remediation by identifying the ranges common to normal, non-water-damaged buildings without visible mould.

To this end, a testing protocol previously reported by Aktas et al. [11, 12] was used, which showed that surface and air sampling should be combined to identify local mould problems, and that an active (aggressive or activated) air sampling strategy, i.e. sampling actively mixed air, better represents indoor mould concentrations than a passive sampling approach, i.e. sampling still air. In this study, the surface and air mould concentration values were measured by means of the quantification of N-acetylhexosaminidase (EC 3.2.1.52; NAHA) activity, expressed in relative fluorescence units (RFUs). This method’s strength lies in that it depicts all fungal propagules (spores, hyphae) [26] and microfragments [27], making it a reliable marker of the fungal biomass present in a given environment. While NAHA is not specific to fungi and can be produced by bacteria and protozoa, and even mammalian cells, making the presence of pollens and pets or number of people using the environment to be tested potentially influential on the readings, this effect has been shown to be negligible [28, 29]. Furthermore, NAHA presence has been found correlated to the non-soluble fraction of β-glucan, and therefore can be used to assess toxicity [30] – although this is beyond the scope of this study. This method has been previously verified by the US Environmental Protection Agency (EPA) Environmental Technology Verification Program [31], and included in the Danish Building and Urban Research Institute instructions [32, 33], as well as ASTM 7338-14 [34].

## Materials and methods

In this study, a total of 140 non-water-damaged rooms in England with no indication of potential moisture induced problems (e.g. damp patches, discolouration, spalling) and no visible mould were tested to identify normal background levels of mould and to benchmark when an indoor environment may be in need of remediation. 140 air samples and 629 surface samples were collected (313 from visually clean surfaces and 316 visually dirty/dusty surfaces).

### Study site

The buildings used for this study include houses (17%), flats/apartments (59%), and single storey detached home (24%) of different ages (pre-1945: 3%, 1946–1970: 70%, 1971–1990: 24%, 1991-today: 3%), and different materials and construction techniques (solid brick masonry, brick masonry with cavity walls – with and without insulation –, concrete) made available to us by volunteers from London and the surrounding counties in England: Huntingdonshire, Cambridgeshire, Soke of Peterborough, Leicestershire, the West Midlands, Staffordshire, Hertfordshire, Essex, Northamptonshire, and Berkshire. In these buildings, 140 non-water-damaged rooms with no visible mould were tested using a testing protocol composed of surface sampling and active (aggressive or activated) air sampling as detailed below.

### Surface sampling

Surface samples were taken using a sterile swab wetted in sterile saline to enhance swabbing efficacy. A 3 × 3-cm adhesive template was used to demarcate the swabbing area. In each room, three to five samples were taken from both visually clean and visually dirty/dusty (but not visibly mouldy) surfaces.

### Active (aggressive or activated) air sampling

Once the air sampling equipment was set up in the room, a handheld blower (Makita BUB 182, 18 V, 0.043 m^3^/s; Makita Corporation, Anjo, Aichi, Japan) was used to disturb all surfaces in the room twice from a distance of approximately 2 m using maximum power on the blower. Air sampling was started 1 min after the blowing phase (to allow large particles to settle), using a flow rate of 15 l/min for 15 min (225 litres total volume), through a cassette preloaded with a 25 mm, 0.8-μm pore size Mixed Cellulose Ester filter (Zefon International, Inc., Ocala, FL, USA). The filters were placed 1.5 m above the ground with the open face filter pointing upwards.

### Mould quantification

Mould concentrations in both surface and air samples were quantified by measuring the activity of β-N-acetylhexosaminidase (NAHA) according to a standardised protocol (Mycometer A/S, Hørsholm, Denmark). Firstly, an enzyme substrate containing 4-methylumbelliferyl was added to the filter or swab samples. After around 30 min of reaction time (depending on ambient temperature), the resulting fluorescence was measured in RFU using a manual fluorometer (Turner Designs, CA, USA/Mycometer version), and the substrate blank value subtracted. One RFU is equal to 33.3 × 10^−2^ pmol 4-MU/ml reaction volume/min. The sampling area for the surface samples was 9 cm^2^ and the reaction volume used for analysing surface samples was 2 ml. The sample volume for air samples were 225 l and the reaction volume for air samples was 1 ml.

### Sample filtering

The obtained air and surface samples were then filtered using visual assessment and microscopy analysis, as described below.

#### a. Visual assessment

Because there is not an objective definition for a clean surface, an additional visual assessment step was carried out to categorise the clean surface samples based on their cleanliness from 0 to 3 from the cleanest to the dirtiest. Only cleanest swabs (category 0, i.e. no stain or discolouration at all) were used for benchmarking when a surface was to be considered clean. The visual assessment was carried out by the same analyst throughout in order to ensure consistency.

#### b. Microscopy analysis

Although all samples were taken from non-water-damaged rooms with no visible mould, because mould growth is not always visible, all surface and air samples which gave a heightened NAHA activity reading were examined under microscopy. To this end, 1 ml of sterile demineralised water was added to the filter after NAHA activity was determined. The filter (still situated in the filtration chamber) was then vortex-mixed for 30 s to release mould from the filter surface and a sample was then taken for microscopy. The microscopy analysis of the sample was performed by the same analyst throughout in order to ensure consistency. All samples that were found to contain hyphae, conidiophores, or a high proportion of mould spores were eliminated from the benchmarking process. In cases where the high NAHA readings were found to be associated with non-mould origin (e.g. skin cells, different types of fibres, and pollen with little mould spores, and other debris due to poor cleaning standards), the samples were included in the benchmarking.

In addition to this, data previously obtained using the exact same testing protocol from 17 buildings in Copenhagen were used here for comparison purposes^1^1These data have been collected by Mycometer A/S between 2008 and 2011, and not been published elsewhere but partly form the basis of the current Danish national benchmarks. They have been granted to this paper for comparison purposes by Mycometer A/S’s Dr Morten Reeslev, a co-author here.. The testing was done exclusively in Copenhagen and led to 86 air samples, 164 samples from visually clean surfaces and 167 samples from visually dirty/dusty surfaces from strictly non-residential locations including the Geology Museum, the Zoology Museum, University of Copenhagen’s Pharmaceutical College, Institutes of Zoology, Botanic, and Molecular Biology, as well as schools and a hospital.

## Results and discussion

### Surface sampling results

The identification of normal background surface mould concentrations was undertaken in two stages: (1) surface samples taken from visually clean surfaces were used to benchmark the upper threshold for the level of mould concentration to target following a cleaning/remediation work, and (2) surface samples taken from visually dusty/dirty surfaces were used to establish the lower threshold for when a surface is likely to have mould growth, and therefore should be considered for further investigation, and possibly remediation.

In order to identify when a surface is to be considered clean, firstly, 313 samples obtained from visually clean surfaces were filtered through visual assessment and microscopy as explained in Materials and methods section. All samples that were not found to be perfectly clean or were established via microscopy to contain mould growth (n = 54) were eliminated, and the remaining 259 samples were used for benchmarking.

In the next stage, in order to identify when a surface is to be considered in need of remediation, those 316 samples from visually dirty/dusty surfaces with heightened NAHA readings were filtered through microscopy analysis. All samples that were found to contain mould growth (n = 18) were eliminated and the remaining 298 samples from visually dirty/dusty surfaces that were found not to include any mould growth were used for benchmarking.

### Air sampling results

For the purposes of benchmarking normal background air mould levels, a similar approach was adopted. Of the total 140 air samples collected, all that demonstrated a heightened NAHA activity were examined via microscopy and eliminated if they showed hyphae, conidiophores, or very high spore counts (n = 10). The remaining 130 air readings were used for air benchmarking.

**Figure 1:**
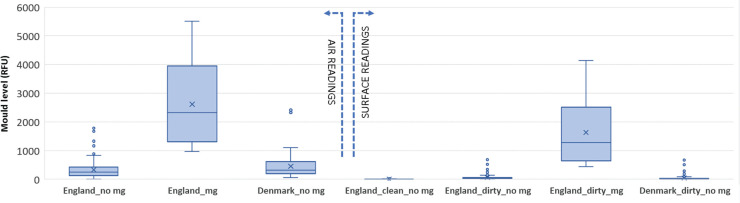
Statistical description of the obtained data (mg stands for mould growth).

A statistical description of the data which were chosen for use in the identification of normal background levels of mould as such is given in Fig. 1 along with the readings from samples found to have mould growth and data from Denmark for comparison purposes.

**Figure 2:**
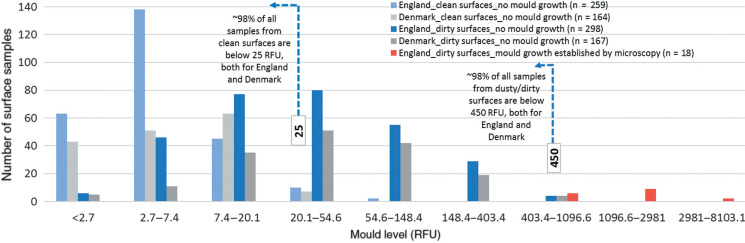
Surface mould levels (x-axis is exponential).

The distribution of the readings from the 259 and 298 samples, respectively, from the visually clean and dusty/dirty surfaces with no mould growth (blue columns) is shown in Fig. 2, along with the readings obtained from 164 and 167 samples with no mould growth collected using the same testing protocol in Denmark (grey columns). The data show that around 98% of all samples from clean surfaces are below 25 RFU, and around 98% of all samples from dusty/dirty surfaces are below 450 RFU, both for England and Denmark. In addition, results from an additional 18 readings that were established by microscopy analysis to have mould growth are also shown in red columns for comparison purposes.

Some of the important conclusions and suggestions based on these observations are summarised:

1. For the set of buildings tested here, 25 RFU is the higher bound for a visually clean surface from a room with no visible mould growth or water damage, and could be used as a success criterion for cleaning and remediation.
2. NAHA activities above 450 RFU are most likely beyond the naturally occurring levels of accumulated mould propagules found on dusty or dirty surfaces in rooms with no visible mould growth or water damage. 450 RFU is therefore the lower bound for a visually dusty/dirty surface from a room with no visible mould growth, and could be used to flag the need for further investigation, and possibly remediation.
3. There is a clear differentiation between samples obtained from surfaces with no mould growth (blue and grey columns), and those that were established to have mould growth via microscopy (red columns). This zone beyond 450 RFU should be further examined by future research to study the distribution of data obtained from indoor environments with different levels of mouldiness.
4. The surface benchmark values that we identified here are highly comparable to those currently in use by the Danish Building Research Institute for more than 15 years as part of the Danish Mould Guidelines [32, 33]. A benchmark of NAHA activity levels ≤25 RFU (referred to as Category A by the Danish Building Research Institute; see [32]) has been used as a success criterion for the cleaning/remediation of surfaces containing mould growth. Similarly, a reading >450 RFU (defined as Category C, [32]) is accepted as the criterion by which to define when to remediate a surface. Category B, defined as readings between 26 and 450 RFU, on the other hand, comprises typical mould concentration levels on dusty/dirty surfaces without mould growth [32].

**Figure 3:**
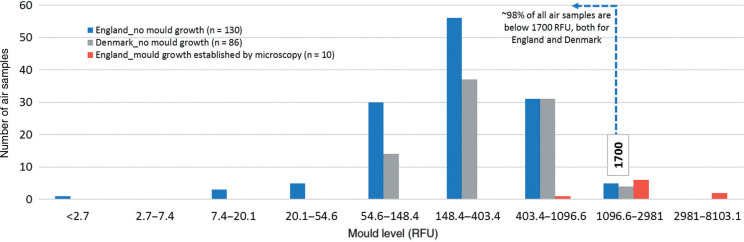
Air mould levels (x-axis is exponential).

The distribution of the readings from the 130 air samples deemed to be suitable for the benchmarking exercise (blue columns) is shown in Fig. 3 along with the readings obtained from 86 air samples taken in Denmark (grey columns). The data show that approximately 98% of all air readings were below 1700 RFU, both for England and Denmark. In addition, results from 10 samples which were established by microscopy that did have mould growth are shown in red columns.

Based on these data we suggest that air values higher than 1700 RFU are most likely indicative of mould growth and might be considered for further examination, and potentially for remediation. Air values lower than 1700 RFU can be further divided into three indicative categories:

1. The lowest one third of all readings (≤200 RFU) could work as the success criteria for a cleaning/mould remediation work.
2. Between 33% and 90% of all readings (201–750 RFU) could be considered the normal background levels of mould within rooms with a good to normal cleaning standard.
3. Between 90% and 98% of all readings (751–1700 RFU) could indicate rooms with a poor to bad cleaning standard, therefore if a reading within this range is obtained from a room with a high cleaning standard, it could be suggested that the room should be checked thoroughly for a possible hidden mould source.
4. Only around 2% of the all readings were >1700 RFU, meaning this is an unlikely outcome from indoor environments with no water damage or visual mould growth, indicating potential for mould growth from an indirect source (hidden mould growth, e.g. in building cavities), or an extremely poor cleaning standard.

The air benchmarks have not yet penetrated into the guidelines by the Danish Building Research Institute, but there are plans in that direction, including the above-mentioned three categories, which may be slightly different in the Danish case. Based on the striking comparability between the English and Danish data, three important observations can be made:

1. Earlier work suggests that mould levels within a given indoor environment are, among many other factors, a product of prevalent outdoor climatic conditions (e.g. for a species focussed discourse see [35]) and how the indoor environment is used, i.e. lifestyle characteristics including heating, cleaning and ventilation habits (e.g. [36, 37]). While the extent of these relations are far from conclusive, the stark similarity between the values obtained for English and Danish properties may suggest that these factors might not be as important as initially thought, or at least that the nuances between England and Copenhagen in terms of these factors are not strong enough to lead to sizable changes in the typical indoor mould levels in these locations (cf. [38]). This should however be further examined by future research. It should also be established whether or not our suggested normal background levels of mould can be extended to the rest of the UK.
2. While all properties tested in England were strictly residential, the data used for the Danish study were obtained from a number of non-residential properties. That these data yielded highly comparable results to England suggests that the function of the indoor environment might not play an important role in the typical indoor mould levels as widely thought. This should however be further examined by future research.
3. While the close similarity between ranges and overall distribution of values obtained from Copenhagen and England in different times of the year (spring/winter and all year round, respectively) may suggest some level of disassociation of the normal background levels of indoor mould from seasons, this should be further examined by future research.

## Conclusions

A lack of reproducibility is one of the major issues in mould research and is largely due to a lack of standardisation of sampling equipment, sampling protocols and analysis methods. However, even when all these are standardised, it is not easy to interpret the obtained readings. In this study, a standard mould measurement method based on the quantification of the activity of N-acetylhexosaminidase (NAHA) was used for surface and active (aggressive or activated) air testing. The distributions of surface and air readings that were obtained from non-water-damaged rooms with no visible mould and no mould growth were studied in order to define normal background levels of mould and benchmark when a surface/indoor environment should be considered clean, and when it should be considered in need for further examination, and potentially remediation.

Our suggested benchmarks are as follows:

- Surface
  0–25 RFU: This level of mould concentration is found on visually clean surfaces within non-water-damaged indoor environments, and could be targeted following a surface cleaning/remediation.
  26–450 RFU: This level of mould is higher than levels typically found on visually clean surfaces, and represents the level measured on dusty/dirty surfaces with no mould growth.
  >450 RFU: This level of mould indicates surfaces with concentrations higher than levels typically found on dusty/dirty surfaces in non-problem indoor environments, and is likely to indicate mould growth. Future work should be undertaken to explore this range further and to study various levels of mould growth and contamination.
- Air
  ≤200 RFU: This level of air mould concentration is typically found in rooms with no visible mould, with a high cleaning standard, and could be targeted following a remediation.
  201–750 RFU: This level of mould in the air is the level found in non-problem, non-water-damaged indoor environments, typically with a normal cleaning standard.
  751–1700 RFU: This level of mould is higher than levels typically found in non-problem rooms with normal cleaning standards. If encountered in a room with very high cleaning standards, this category may mean that the room is in need of further investigation.
  >1700 RFU: This level of mould is higher than levels typically found in non-problem indoor environments, and is likely to indicate mould growth. Future work should be undertaken to explore this range further and to study various grades of mould growth and contamination.

The benchmarks here are specific to the testing method and protocol used. However, the same approach can be adapted to any other mould testing technology with a clear testing protocol. The association between our suggested thresholds and potential health implications should be explored by further research, including also speciation, and different levels of mould growth and contamination.

Finally, the similarity of benchmarks defined for England and Denmark may suggest that the some of the factors that are currently accepted to govern typical indoor mould levels – primarily the climatic conditions and lifestyle – are either not as influential, or they are not sufficiently different between these two countries to lead to a substantial differences in the findings.

The benchmarks suggested here are not rigid, and aim to provide some insight into the testing values. These preliminary results will be further developed by future research to delve into the impact of seasons, functions, local climatic conditions and different levels of mouldiness, and be extended to multiple methods.

## Data Availability

The datasets generated during and/or analysed during the current study are available from the corresponding author on reasonable request.
